# IBD-related inflammatory memory and colorectal cancer: epigenetic mechanisms and microbiome interventions

**DOI:** 10.3389/fimmu.2026.1912351

**Published:** 2026-08-19

**Authors:** Guojun Gao, Xin Zhao, Xingyu Lu, Shuyue Yu, Lihua Chen

**Affiliations:** 1 School of Medical Technology, Shaanxi University of Chinese Medicine, Xianyang, Shaanxi, China; 2 Department of Immunology, School of Basic Medicine, Air Force Medical University, Xi’an, Shaanxi, China

**Keywords:** colitis-associated cancer, epigenetic regulation, gut microbiota, immune microenvironment, inflammatory memory, precision prevention

## Abstract

Colitis-associated cancer (CAC) develops within chronically inflamed mucosa and differs from sporadic colorectal cancer in its field effects, multifocality, and sequence of molecular events. In addition to ongoing inflammation and mutation, experimental studies indicate that epithelial, immune, and stromal compartments can retain altered states after an initiating inflammatory stimulus has subsided. In this review, inflammatory memory is used operationally for a persistent molecular, cellular, tissue, or microbial state that changes the response to a later challenge. This definition distinguishes epithelial epigenetic memory from trained innate immunity, adaptive lymphocyte memory or exhaustion, and chronic signaling that depends on continued stimulation. We synthesize evidence for persistent chromatin accessibility, histone modification, DNA methylation, enhancer activity and three-dimensional organization, epithelial plasticity, immune–stromal circuits, and microbiota-derived metabolites. These responses can support mucosal repair, but repeated activation within a genetically altered field may facilitate clonal expansion and tumor development. We also assess emerging methylation, circulating tumor DNA, stool DNA, single-cell, and spatial biomarkers and grade proposed interventions according to evidence from cell culture, organoids, animal models, human tissues, and clinical studies. Because chromatin- and microbiome-directed interventions remain largely preclinical, selective modulation of pathological persistence—not complete “memory erasure”—is the appropriate translational objective.

## 1 Introduction

Colorectal cancer (CRC) remains a major global cause of cancer morbidity and mortality, and chronic intestinal inflammation is an established independent risk factor. The global expansion and accumulated prevalence of inflammatory bowel disease (IBD), principally ulcerative colitis (UC) and Crohn’s disease (CD), increase the number of patients requiring long-term cancer prevention (1, 2, 15–18). Patients with long-standing, extensive, or poorly controlled IBD carry increased risk of colitis-associated cancer (CAC), particularly when additional factors such as primary sclerosing cholangitis, family history, strictures, or prior dysplasia are present (4, 13, 19, 20, 29, 51). In contrast to sporadic CRC, CAC tends to arise in a diffuse inflammatory field, may be multifocal or flat, and can be difficult to detect endoscopically. These clinical features motivate a mechanism-focused account of how prior inflammation may continue to influence tissue behavior during apparent remission.

CAC is not simply sporadic CRC occurring in a patient with IBD. It usually develops through an inflammation–dysplasia–carcinoma sequence in which genetically and epigenetically altered clones can occupy broad areas of histologically non-neoplastic mucosa (4, 26, 28, 45, 66, 96). Early TP53 alterations, later and less frequent APC disruption, microbial genotoxicity, epithelial plasticity, immune remodeling, and stromal changes can therefore interact across the mucosal field (6, 12, 26, 49, 73, 96). This review concentrates on those persistent interactions and their implications for risk assessment and prevention.

Here, inflammatory memory is defined operationally as a molecular, cellular, tissue, or microbial state induced by inflammation that persists after the initiating signal has substantially declined and changes the response to a later challenge. It is distinct from ongoing chronic signaling, which requires continued stimulation. Epithelial epigenetic memory refers to persistent chromatin or transcriptional competence in epithelial lineages; trained immunity describes altered recall responses in innate immune cells; adaptive immune memory denotes antigen-specific lymphocyte persistence; and T-cell exhaustion is a distinct, often partially stable state caused by prolonged antigen exposure (14, 38, 78, 103, 104). Stromal matrix remodeling and persistent microbial community remodeling are included only when they remain after the initiating inflammatory condition has subsided.

The specific perspective of this review is to examine CAC pathogenesis through persistence after inflammatory resolution: what persists, which cell or compartment carries the state, whether it is reversible, and how it could be measured or safely modified. This persistence-centered synthesis connects epithelial chromatin, immune and stromal remodeling, and microbiota without treating any one pathway as a universal driver (50, 69, 96). Because complete reversal has not been demonstrated clinically, the terms “memory modulation,” “reversal,” or “interception” are used instead of treating “memory erasure” as an established therapeutic outcome.

## 2 From mutational accumulation to inflammatory field cancerization

### 2.1 The canonical vogelstein model versus the inverted genetic sequence of CAC

CRC develops through several evolutionary routes. In the conventional sporadic adenoma–carcinoma model, early APC loss and Wnt-pathway activation are followed by additional alterations that can include KRAS activation, 18q loss involving SMAD4 and deleted in colorectal carcinoma (DCC), and TP53 inactivation; the order is not invariant (13, 30–33). Morphologically, this model is associated with progression from normal mucosa through a polypoid adenoma to invasive carcinoma, creating an opportunity for colonoscopic detection and polypectomy (4, 21, 26, 34, 35).

CAC more often follows an inflammation–dysplasia–carcinoma sequence. TP53 mutation or loss of heterozygosity can occur in non-dysplastic IBD mucosa, whereas APC alterations tend to occur later and less frequently than in sporadic CRC (5, 6, 12, 26, 36). The associated field effect helps explain why dysplasia may be flat, multifocal, and poorly demarcated (5, 37). These tendencies are clinically important but are not universal: the order, clonality, morphology, and tempo of progression vary among patients and lesions (38–41, 96).

### 2.2 Field cancerization and early clonal diversification

Genomic and phylogenetic studies support extensive field cancerization in IBD, although the term “Big Bang” should not be taken to imply that every CAC follows one abrupt evolutionary pattern (25, 42, 96). Repeated injury can impose selective pressure across an entire mucosal segment, allowing multiple altered clones to expand before dysplasia becomes histologically apparent. The resulting cancers may therefore be multifocal and genetically heterogeneous, but the timing and clonality of individual lesions remain patient- and model-dependent.

**Figure 1:**
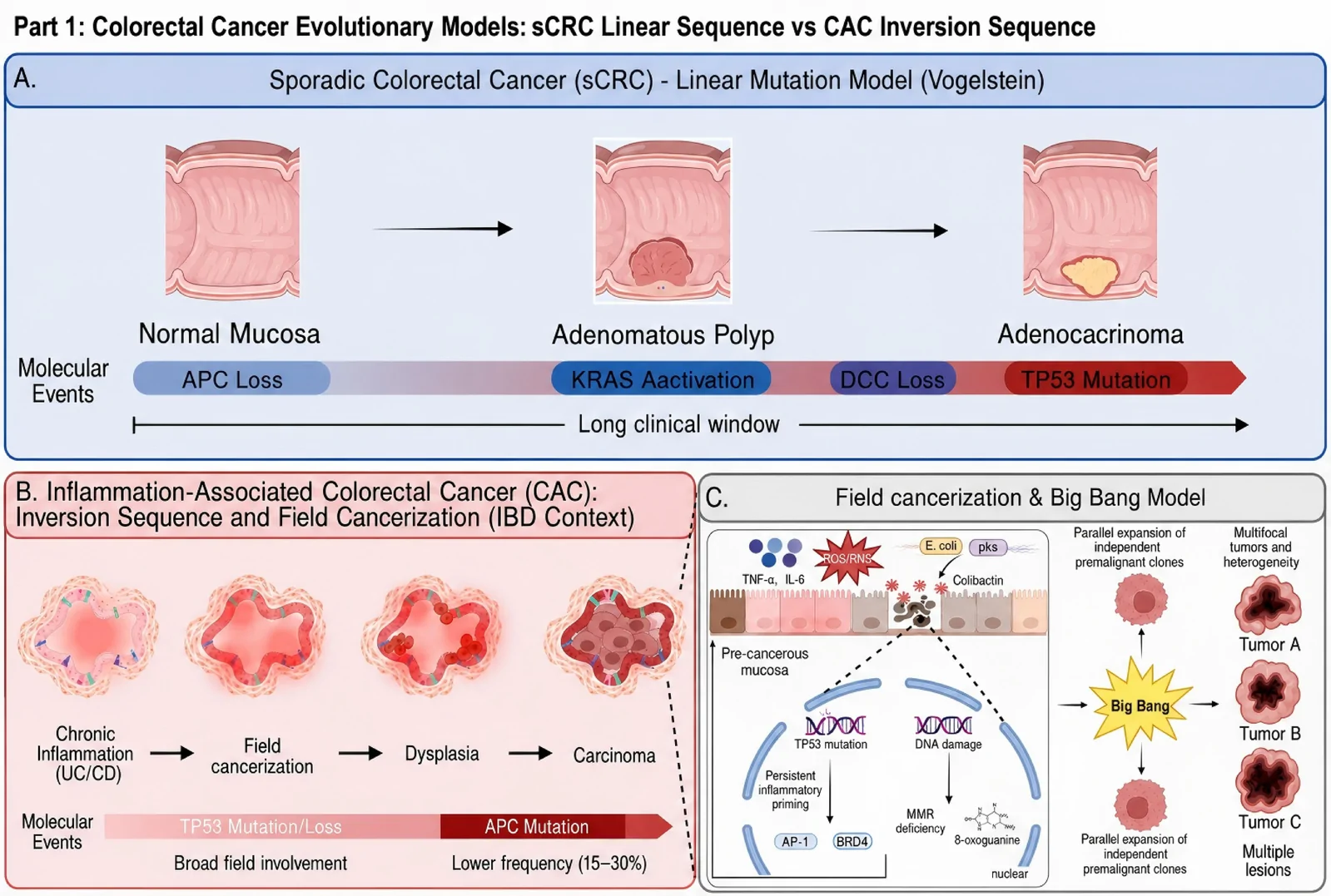
Genetic evolution and field cancerization in CRC. **(A)** The sporadic CRC sequence is summarized as an established model. **(B)** Earlier TP53 alteration and later, less frequent APC disruption in CAC are supported by human molecular studies, although individual tumors vary. **(C)** The integration of inflammation, ROS, microbial genotoxins, clonal diversification, and persistent chromatin changes is a synthesis of established components; the single “Big Bang” depiction is a conceptual model rather than a universal temporal sequence.

Field cancerization develops through interacting layers rather than a single event. Cytokines, reactive oxygen species (ROS), and reactive nitrogen species (RNS) promote DNA damage and impaired repair, while genetically altered but histologically bland clones can expand under inflammatory selection (25, 30, 44, 96). TP53- or KRAS-mutant patches have been detected in non-neoplastic IBD mucosa, and independent clones may subsequently progress in parallel (6, 47, 96). This framework helps explain multifocality and supports surveillance of the entire at-risk colon rather than only previously visible lesions; however, it does not establish that all inflamed mucosa is irreversibly transformed. The contrasting evolutionary routes of sporadic CRC and CAC, together with the field-cancerization framework, are summarized in **Figure 1**.

### 2.3 The molecular linkage driving field cancerization: from oxidative stress to epigenetic remodeling

Microbial genotoxicity is one component of inflammatory field cancerization. Colibactin-producing pks-positive *Escherichia coli* (pks+ *E. coli*) can generate DNA double-strand breaks (DSBs) and a characteristic mutational signature, whereas enterotoxigenic *Bacteroides fragilis* can promote barrier disruption and inflammatory signaling through B. fragilis toxin (44, 53–55, 102). Host-derived oxidative and nitrosative stress can compound this damage. In experimental systems, limiting inducible nitric oxide synthase or oxidative injury reduces microbe-associated tumorigenesis, but these findings do not by themselves establish antioxidant chemoprevention in patients (24, 30, 44).

At the epigenetic level, inflammatory signaling through NF-κB, STAT3, and activator protein 1 (AP-1) can alter enhancer accessibility and transcriptional responsiveness (42, 56, 58, 59). Direct mouse evidence shows that AP-1-associated chromatin accessibility can persist after inflammatory injury, whereas bromodomain-containing protein 4 (BRD4) is one acetyl-lysine reader within a broader regulatory network rather than a solitary master regulator (14, 59, 78). Histone acetylation and methylation, DNA methylation, Polycomb activity, enhancer RNAs, and chromatin looping may reinforce or counteract these states. Human IBD-derived intestinal stem-cell organoids retain parts of an inflammation-associated epigenetic program ex vivo, and experimental studies independently support inflammation-induced stem-cell imprinting (103, 104). Longitudinal validation in human CAC nevertheless remains limited. Thus, epigenetic persistence is best considered a graded, cell- and context-dependent contributor to field cancerization.

## 3 Core mechanisms: how inflammation imprints “cellular memory”

### 3.1 Cellular carriers, functional consequences, and reversibility

Memory-like persistence is unlikely to be uniform across the inflamed colon. Epithelial stem and progenitor cells can carry chromatin and DNA-methylation states through cell division; fibroblasts may preserve transcriptional and extracellular-matrix programs; macrophages may acquire trained or tolerant innate responses; and T cells may retain antigen-specific memory or exhaustion-associated chromatin (9, 14, 38, 60, 65, 78, 80, 103, 104, 115). Microbial communities may also retain altered composition or function after inflammation subsides, but this ecological persistence is not cellular epigenetic memory.

Reversibility depends on the carrier and readout. Cytokine withdrawal may normalize short-lived transcription, differentiation can dilute some epithelial states, matrix turnover can remodel a fibroblast-generated niche, and immune states can be reprogrammed incompletely. Conversely, DNA methylation, Polycomb-associated repression, enhancer topology, or stable clonal selection may persist. **Table 1** separates these compartments and indicates where evidence is direct, indirect, or still inferential.

**Table 1:** Cell- and compartment-specific forms of inflammatory persistence and their reversibility.

Compartment	Candidate carrier/form	Potential consequence	Reversibility	Current evidence
Epithelial stem/progenitor cells	Chromatin accessibility; histone marks; DNA methylation; lineage composition	Faster repair response or expansion of altered clones	Partial; depends on differentiation and mark stability	Direct in mouse injury models; human IBD organoid retention; limited longitudinal human CAC data (14, 103, 104)
Fibroblasts/extracellular matrix (ECM)	Persistent transcriptional state; collagen organization; matrix stiffness	Oncostatin M (OSM)/C-C motif chemokine ligand 2 (CCL2) signaling; Yes-associated protein/transcriptional coactivator with PDZ-binding motif (YAP/TAZ) activation; altered immune access	Potentially reversible through cell turnover and matrix remodeling	Human single-cell association; causality strongest in experimental systems (60, 80)
Macrophages/innate myeloid cells	Trained or tolerant metabolic and chromatin programs	Amplified inflammation, repair, immunosuppression, or impaired recall	Context-dependent and often partial	General trained-immunity evidence; CAC-specific persistence incompletely tested (9, 38)
T lymphocytes	Antigen-specific memory; exhaustion-associated chromatin	Surveillance, tolerance, or dysfunctional cytotoxicity	Memory can persist; exhaustion may be only partly reversible	Human IBD/CRC heterogeneity; scarce longitudinal CAC studies (65, 70, 115)
Microbiota	Persistent community composition and metabolic networks	Genotoxin exposure or protective metabolite production	Modifiable but often unstable and host-dependent	Mouse causality for selected pathways; human evidence largely associative (12, 22, 54)

### 3.2 AP-1/BRD4 within a multilayered chromatin network

A central feature of the proposed framework is persistence after the initiating stimulus has declined. In intestinal epithelium, this persistence may reside in altered chromatin accessibility, histone modifications, DNA methylation, enhancer–promoter communication, or stable shifts in stem/progenitor composition (14, 58, 59, 78, 103–105). These mechanisms should not be equated with active inflammation that simply continues unabated; persistence must be demonstrated after removal or resolution of the original signal.

AP-1, composed of JUN and FOS family proteins, can bind stress-responsive regulatory regions and contribute to enhancer opening. In experimental intestinal injury, persistent AP-1-associated accessibility provides direct support for an epithelial memory mechanism (14, 58, 59). BRD4 can read acetylated lysines and support transcription at active enhancers, but its participation does not establish an exclusive AP-1/BRD4 “lock.” Histone acetyltransferases and deacetylases, methyltransferases and demethylases, ATP-dependent remodelers, and lineage-specific transcription factors jointly determine whether a regulatory state is maintained.

Repair-associated genes, including IL-33 and REG-family genes, may remain more readily inducible after injury, thereby increasing the magnitude or speed of a subsequent response (56, 58, 63, 94). Such priming can aid barrier restoration, but repeated activation in a field carrying TP53, KRAS, or DNA-repair defects may favor survival and expansion of altered clones. Accordingly, enhancer persistence is presented here as a conditional susceptibility state, not as proof that all clinically healed mucosa is constitutively pre-malignant or irreversibly committed to cancer.

Beyond enhancer acetylation, DNA methylation drift has been detected in colitis-associated neoplasia and has produced candidate risk markers (101, 105). Histone methylation and Polycomb complexes, including enhancer of zeste homolog 2 (EZH2)-containing Polycomb repressive complex 2 (PRC2), can stabilize lineage or inflammatory programs, whereas histone deacetylases (HDACs) can either restrain or promote inflammation depending on isoform and cell type (78, 97, 98). Enhancer RNAs, chromatin looping mediated by CCCTC-binding factor (CTCF) and cohesin, and three-dimensional enhancer–promoter contacts are plausible persistence mechanisms, but direct longitudinal evidence in human CAC is sparse. This broader architecture argues against assigning master-regulator status to AP-1/BRD4.

### 3.3 Epithelial plasticity and potential non-LGR5 origins

LGR5-positive (LGR5+) intestinal stem cells are a well-established cell of origin in many experimental colorectal tumors. CAC specimens often show reduced or absent LGR5 expression, but loss of a marker in an established tumor does not by itself prove a non-LGR5 cell of origin (36). Lineage-tracing experiments in inflamed mice provide stronger evidence that differentiated secretory lineages can dedifferentiate and initiate tumors in appropriate genetic contexts (99). Whether the same lineages dominate human CAC remains unresolved.

Inflammation-induced plasticity can broaden the population capable of acquiring stem-like behavior through NF-κB, STAT3, and wound-repair programs. Candidate progenitor-associated markers include CD133 and special AT-rich sequence-binding protein 2 (SATB2), but these markers identify cell states rather than uniquely defining lineage ancestry (36, 99). Together, these data indicate that CAC may arise through multiple, context-dependent cellular routes, including conventional stem cells and inflammation-enabled dedifferentiation.

### 3.4 Metabolic windows and context-dependent regeneration

Metabolic state can modulate intestinal regeneration, but evidence must be separated from chronic IBD. Fasting followed by refeeding can transiently increase stem-cell activity in experimental systems, providing a useful model of how nutrient-responsive pathways intersect with repair (76). It does not, by itself, demonstrate a clinically relevant fasting–refeeding mechanism in human CAC.

**Figure 2:**
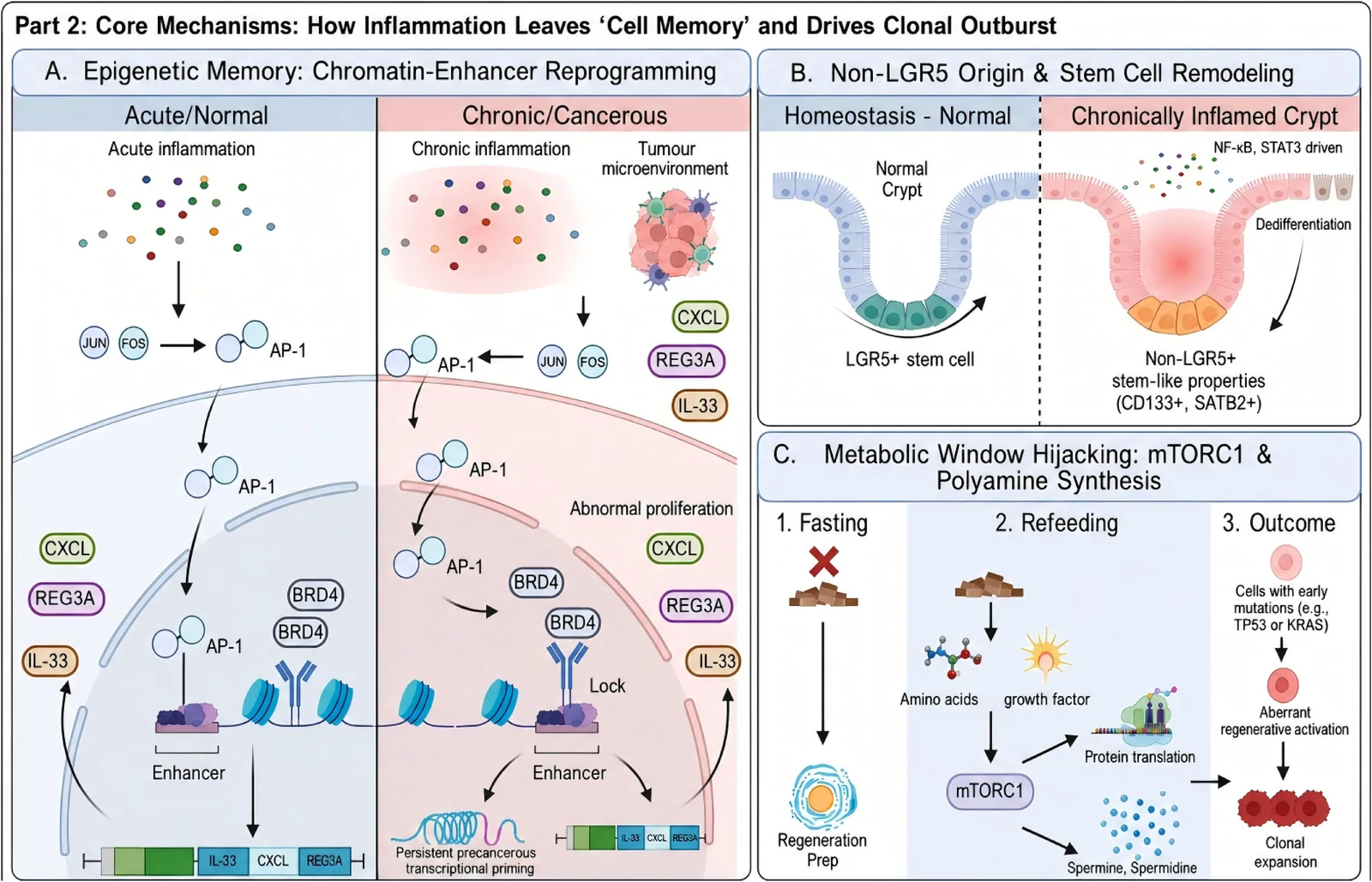
Candidate mechanisms of epithelial inflammatory persistence and clonal expansion. **(A)** Persistent AP-1-associated enhancer accessibility is supported by mouse colitis experiments; BRD4 is shown as one component of a broader chromatin network. The ‘Lock’ label in the schematic denotes persistent transcriptional priming rather than irreversible chromatin fixation. **(B)** Inflammation-enabled dedifferentiation is supported by lineage tracing in mice, whereas a universal non-LGR5 origin in human CAC remains hypothetical. **(C)** Fasting–refeeding and polyamine effects derive mainly from non-chronic-colitis experimental systems and are presented as a testable metabolic interaction rather than an established human CAC mechanism.

mTORC1 signaling and polyamine metabolism can increase translation and proliferation during regenerative responses (55, 76). In a genetically or epigenetically altered field, the same response could facilitate clonal expansion; in an intact epithelium, however, it may support physiological repair. This duality illustrates why metabolic “memory activation” remains a hypothesis requiring validation in chronic colitis models, patient-derived organoids, and prospective human cohorts. The proposed links among epigenetic persistence, inflammation-enabled stem-like plasticity, and metabolically triggered clonal expansion are summarized in **Figure 2**.

### 3.5 Bidirectional coupling with the immune and stromal microenvironment

Persistent epithelial states are shaped by reciprocal signals from immune and stromal compartments. Oncostatin M (OSM) binding to the OSM receptor (OSMR) can activate STAT3 and is associated with severe IBD and resistance to anti-TNF therapy (56, 57, 77). These findings support an OSM-centered inflammatory circuit, but direct evidence that OSM permanently “locks” epithelial chromatin in human CAC is not yet available. Cancer-associated fibroblasts (CAFs), extracellular matrix, and myeloid cells may sustain the circuit through cytokine production and mechanotransduction.

Prior inflammatory exposure can also alter responses to microbial products, but identical toll-like receptor 4 (TLR4) ligands may produce different outcomes depending on cell lineage, mutation status, and local metabolites (27, 44, 52, 78, 79). Some persistent programs diminish after withdrawal of cytokines, restoration of the niche, differentiation, or pharmacological chromatin modulation, whereas others remain through cell division. Reversibility is therefore an empirical property to be measured—not an assumption—and may differ among epithelial, myeloid, lymphoid, stromal, and microbial compartments.

Importantly, inflammatory memory is not intrinsically tumor-promoting. A primed epithelial or innate response can accelerate barrier repair and antimicrobial defense. Risk is expected to rise when the response is excessive, repeatedly reactivated, coupled to mutational selection, or maintained in an immunosuppressive and mechanically altered niche. This context dependence is central to therapeutic design because indiscriminate removal of repair competence could worsen mucosal injury.

## 4 The driving forces: cytokine storms and metabolic reprogramming

### 4.1 The double-edged sword of IL-22: from tissue repair to tumorigenesis

Interleukin-22 (IL-22) is an important regulator of epithelial repair. It is produced by type 3 innate lymphoid cells (ILC3s), T helper 22 (Th22) cells, and other lymphocytes and signals through IL-22R1/IL-10R2 to activate JAK1/TYK2 and STAT3. In acute injury models, this pathway induces antimicrobial peptides and mucins and can protect intestinal stem cells (ISCs) (63, 64, 79). Its function nevertheless varies with tissue state, microbial context, and duration of exposure.

During chronic colitis and tumorigenesis, prolonged IL-22–STAT3 signaling can support epithelial survival and proliferation, particularly when negative feedback or tumor-suppressive checkpoints are impaired (42, 52, 63). IL-22-dependent chemokine programs can also recruit neutrophils whose reactive oxygen species and proteases may add tissue damage, although neutrophil states are heterogeneous and can also contribute to microbial control and repair (44, 56, 64, 70). IL-22 should therefore be considered a stage- and context-dependent cytokine rather than a fixed switch from protection to tumor promotion.

### 4.2 Cell-specific immunity and tissue organization

Macrophage states in IBD and CAC occupy a continuum rather than a binary M1/M2 classification. Pro-inflammatory, wound-healing, lipid-associated, and immunoregulatory programs can coexist, and prior stimulation may generate trained or tolerant recall responses (9, 38, 70). Dendritic cells integrate microbial signals and shape regulatory T (Treg) versus effector responses; innate lymphoid cells, particularly ILC3s, provide IL-22 that can support barrier repair yet also sustain transformed epithelium (8, 63, 64, 70). Neutrophils are likewise heterogeneous, with subsets contributing ROS, proteases, angiogenesis, microbial control, or repair (8, 65, 70).

Treg cells can limit tissue-damaging inflammation but may also suppress antitumor immunity once neoplasia is established (8, 42, 70). Chronically stimulated CD8+ T cells can acquire exhaustion-associated chromatin and reduced cytotoxicity, while tertiary lymphoid structures (TLSs) may organize local antigen presentation and are variably associated with favorable immune surveillance (8, 42, 65, 70, 115). The abundance, location, and functional state of these populations differ across active colitis, remission, dysplasia, and invasive CAC; human spatial and longitudinal studies are therefore necessary before assigning a fixed protective or pathogenic role.

### 4.3 OSM/IL-23 signaling and cancer-associated fibroblasts

OSM is produced predominantly by activated myeloid and stromal cells, and OSMR is expressed by stromal and epithelial compartments. OSM–OSMR signaling can activate JAK–STAT3 programs associated with refractory IBD (56, 57, 77). Interleukin-23 (IL-23) can sustain pathogenic lymphoid and myeloid circuits, while cancer-associated fibroblasts (CAFs) provide cytokines, extracellular matrix, and metabolic support. These axes may reinforce epithelial survival, but their persistence and causal contribution to human CAC require spatial and longitudinal validation.

Cytokine-driven induction of activation-induced cytidine deaminase and other DNA-damage pathways offers a potential link between immune signaling and mutagenesis (42, 56, 70). Nevertheless, cytokine abundance, mutational signatures, and fibroblast states vary across patients and disease stages. The OSM/IL-23 network should therefore be viewed as a context-dependent component of the CAC microenvironment rather than a universal, self-sufficient driver.

### 4.4 Host–microbial metabolism: harmful and protective signals

Inflammation can remodel host polyamine metabolism through ornithine decarboxylase 1 (ODC1) and spermine oxidase (SMOX), generating proliferative substrates and reactive by-products (30, 55, 76). In parallel, nitrate and other electron acceptors can favor Enterobacteriaceae and enhance exposure to microbial genotoxins such as colibactin (44, 53–55, 102). These mechanisms are supported mainly by experimental models; metabolite concentrations, dietary context, and microbial strain variation complicate extrapolation to individual patients.

Tungstate can disrupt molybdenum cofactor (Moco)-dependent bacterial respiration and reduce inflammation-associated dysbiosis in mice (54). This is a selective ecological intervention rather than proof of clinical CAC prevention. Moreover, the microbiome also produces potentially protective metabolites: butyrate and other short-chain fatty acids (SCFAs) can support barrier integrity and regulatory immunity; indole derivatives can signal through the aryl hydrocarbon receptor (AhR); and selected secondary bile acids may exert protective or harmful effects depending on concentration, receptor context, community structure, and diet (12, 22, 75, 89–91). Mitochondrial metabolism is another potential interface between inflammation and epithelial fitness, but therapeutic manipulation remains experimental (83). A balanced model must therefore account for both genotoxic and homeostatic microbial functions.

### 4.6 Stromal mechanics and tissue-level persistence

Single-cell profiling has identified fibroblast states associated with IBD remodeling, including populations linked to charged multivesicular body protein 1A (CHMP1A), TBX3, and ring finger protein 168 (RNF168) (60, 80). Fibroblasts can recruit myeloid cells through OSM and CCL2 and increase matrix stiffness through collagen deposition. Whether these particular states constitute stable “memory” in human CAC, rather than ongoing responses to inflammation, remains to be established.

**Figure 3:**
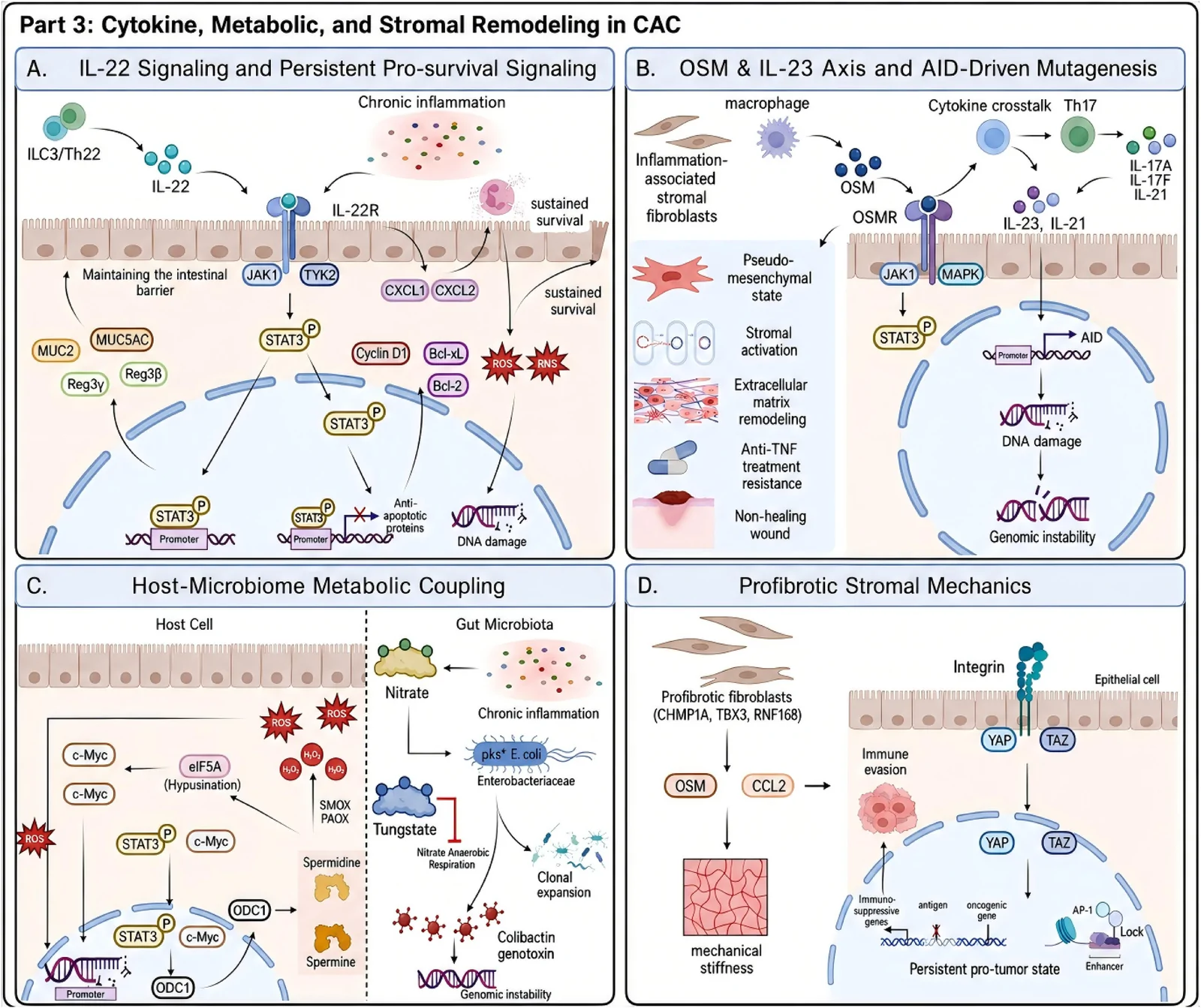
Immune, metabolic, and stromal drivers of CAC. **(A)** IL-22/JAK–STAT3 survival signaling and neutrophil-derived oxidative damage are supported mainly by experimental studies. **(B)** OSM/IL-23 signaling and activation-induced cytidine deaminase (AID)-linked mutagenic pathways are mechanistically plausible but heterogeneous in human tissue. **(C)** Ornithine decarboxylase 1 (ODC1), spermine oxidase (SMOX), and N1-acetylpolyamine oxidase (PAOX) metabolism, nitrate respiration, and tungstate effects are established principally in animal models; human preventive efficacy is unproven. **(D)** Matrix stiffness and YAP/TAZ activation are supported components, whereas a self-sustaining stromal memory loop remains a conceptual synthesis requiring longitudinal validation. The ‘AP-1 Lock’ label denotes a hypothesized primed chromatin state, not a proven irreversible mechanism.

Matrix stiffness can activate integrin–YAP/TAZ signaling in epithelial cells and may restrict immune-cell access (42, 80, 81). This creates a plausible feedback loop among fibroblasts, epithelium, and immunity. However, causal evidence is strongest in reductionist and animal systems; human spatial studies are needed to determine whether stiffness precedes neoplasia, follows chronic injury, or both. The cytokine, host–microbial metabolic, and stromal circuits that may sustain inflammatory persistence in CAC are summarized in **Figure 3**.

## 5 Clinical translation: precision intervention and “niche” editing

### 5.1 Risk stratification: molecular biomarkers for the identification of high-risk patients

Risk stratification should integrate established clinical variables—disease duration and extent, cumulative inflammatory burden, primary sclerosing cholangitis, family history, strictures, post-inflammatory polyps, and prior dysplasia—with high-quality chromoendoscopic surveillance (3, 4, 13, 28, 29, 51, 61, 67, 95). Reported metabolic or genetic associations, including obesity-related signals and candidate DNA-repair factors such as ERCC4, remain exploratory and are not part of validated CAC surveillance algorithms (62, 68). Molecular markers are promising adjuncts, but none currently replaces guideline-based colonoscopy and histopathology (10).

Single-cell RNA sequencing (scRNA-seq) has resolved epithelial, immune, and stromal states in IBD and CRC, including profibrotic fibroblasts and exhausted lymphocyte populations (60, 65, 80, 115). These signatures can generate mechanistic hypotheses and candidate risk scores, but cross-platform reproducibility, sampling depth, treatment effects, and the scarcity of longitudinal pre-dysplasia CAC cohorts limit immediate clinical use.

OSM, OSMR, and IL-23-related transcriptional programs are associated with inflammatory severity and anti-TNF nonresponse (56, 57, 77). Their use as CAC biomarkers remains investigational: association with refractory inflammation does not establish specificity for neoplastic progression, and thresholds have not been prospectively validated for surveillance decisions. They should therefore complement, not determine, endoscopic or therapeutic management.

Emerging diagnostic approaches include plasma circulating tumor DNA (ctDNA), DNA methylation-based markers, multitarget stool DNA assays, and tissue spatial-transcriptomic signatures. Methylation panels have shown feasibility for colitis-associated neoplasia, and serial ctDNA may be useful after a cancer diagnosis; however, low tumor shedding, background clonal hematopoiesis, field-wide methylation changes, and uncertain thresholds limit early-CAC screening (29, 101, 105). Small serum-marker studies are also exploratory and are not specific enough to guide CAC surveillance (72). Stool DNA assays validated for average-risk sporadic CRC cannot be assumed to perform equivalently in actively inflamed IBD, where bleeding and epithelial turnover may alter specificity.

Mechanistic resolution is being accelerated by scRNA-seq, spatial transcriptomics, single-cell assay for transposase-accessible chromatin using sequencing (scATAC-seq), clustered regularly interspaced short palindromic repeats (CRISPR)-based functional screening, and patient-derived intestinal organoids (31, 60, 80, 103, 114, 115). scRNA-seq identifies cell states; spatial methods preserve epithelial–immune–stromal neighborhoods; scATAC-seq maps regulatory accessibility; CRISPR screens test candidate regulators; and organoids assess cell-intrinsic persistence after removal from the inflammatory niche. Each technology also has limitations, including dissociation bias, sparse chromatin coverage, incomplete immune/stromal representation in epithelial organoids, and the need for prospective clinical linkage.

### 5.2 Evidence-graded intervention strategies

Candidate interventions span established clinical prevention, human biomarker studies, animal models, organoids, and *in vitro* systems. **Table 2** identifies the highest current evidence level and separates clinically validated management from experimental memory- or microbiome-directed approaches. A targeted ClinicalTrials.gov search performed on 17 July 2026 did not identify a CAC-specific prevention trial of BET/BRD4 inhibition, HDAC inhibition, tungstate, *A. muciniphila*, engineered probiotics, bacteriophages, or CRISPR-based epigenome editing; trials in other diseases cannot be taken as evidence of CAC prevention.

**Table 2:** Evidence level and translational status of candidate interventions for CAC prevention or risk reduction.

Intervention	Proposed target	Highest evidence	Human CAC status	Principal limitation	Refs.
Inflammation control and surveillance	Cumulative inflammatory burden; early dysplasia detection	Clinical guidelines and observational cohorts	Current standard of care	Does not directly prove reversal of persistent molecular states	(3, 4, 13, 29, 110)
Bromodomain and extraterminal (BET)/BRD4 inhibition	Acetyl-lysine-dependent enhancer transcription	Cell and animal studies; mechanistic inference	No validated CAC-prevention indication	Broad transcriptional effects, repair and hematologic toxicity	(14, 58, 59)
HDAC inhibition	Histone acetylation and inflammatory transcription	Azoxymethane/dextran sulfate sodium (AOM/DSS) mouse models	No validated CAC-prevention indication	Isoform- and cell-specific effects; systemic toxicity	(98)
Cytokine/JAK modulation	OSM/IL-23/JAK–STAT pathways	Approved IBD therapies plus human biomarker studies	Clinical for IBD; not validated as memory reversal	Cancer-specific safety and causal biomarker validation	(56, 57, 77, 110, 111)
Tungstate	Microbial Moco-dependent respiration	Mouse models	No established human CAC efficacy	Dose, off-target effects, ecological durability	(54)
*A. muciniphila*/Amuc_1100	Barrier and immune modulation	Conflicting mouse and associative human data	No established CAC-prevention efficacy	Stage-, strain-, dose-, diet-, and host-dependent effects	(23, 81, 100, 109)
Lactobacillus cocktails	Wnt/Notch and epithelial growth	CRC cell-line studies	Not clinically validated for CAC	*In vitro* findings may not translate to inflamed mucosa	(84)
Engineered probiotics	Defined metabolite or therapeutic delivery	Mouse proof-of-concept	Experimental; no CAC-specific trial identified	Containment, gene transfer, manufacturing, safety	(108)
Bacteriophage therapy	Selective depletion of harmful bacterial strains	Mouse proof-of-concept in CRC	Experimental; no CAC-specific trial identified	Resistance, community effects, delivery, regulation	(107)
CRISPR epigenome editing	Locus-specific transcriptional memory	Cellular proof-of-concept outside CAC	Experimental; no CAC-specific trial identified	Delivery, specificity, durability, reversibility	(106)

Tungstate-mediated microbiome editing has reduced inflammation-associated Enterobacteriaceae fitness and tumor burden in mouse models, including chemically induced and genetically susceptible settings (53, 54). No CAC-specific human efficacy or safety trial has yet established tungstate as a preventive therapy. Dose selection, off-target effects on host and microbial molybdoenzymes, long-term ecological stability, and interindividual microbiome variation remain major barriers.

The effects of *Akkermansia muciniphila* are context-dependent. Barrier-supporting and immunomodulatory effects have been reported, including activity of the membrane protein Amuc_1100, whereas other studies found increased abundance or worsened tumorigenesis in particular colorectal cancer models (23, 81, 100, 109). Disease stage, baseline community structure, strain, dose, diet, and host genotype may determine direction of effect. *A. muciniphila* should therefore not be presented as a uniformly protective CAC probiotic.

Lactobacillus cocktails have inhibited growth and modulated Wnt/Notch signaling in colorectal cancer cell lines, but this is *in vitro* evidence and does not establish prevention in IBD (84). Engineered probiotics and bacteriophages could deliver defined functions or selectively remove harmful strains, yet containment, horizontal gene transfer, phage resistance, manufacturing consistency, and inflamed-mucosa safety require resolution before clinical CAC prevention trials (107, 108).

## 6 Clinical decision-making: therapeutic strategies for coexisting IBD and colorectal cancer

Management of IBD in patients with cancer also depends on the malignancy site, stage, metastatic pattern, and planned anticancer treatment. Cohort and review data address extracolonic malignancies, treatment-associated IBD activity, peritoneal disease, radiotherapy, and bevacizumab, but these heterogeneous observations do not validate an inflammatory-memory intervention (40, 43, 46, 48, 82, 87, 92, 93). Multidisciplinary assessment remains necessary for decisions that extend beyond CAC surveillance and prevention.

### 6.1 Conventional therapies: 5-aminosalicylates and thiopurines

In patients with IBD who have colorectal neoplasia or a previous malignancy, treatment selection should balance control of inflammatory activity against treatment-specific cancer risks (3, 74, 85, 86, 110, 111). Observational and meta-analytic studies suggest that regular 5-aminosalicylic acid (5-ASA) use may be associated with lower colorectal neoplasia risk in ulcerative colitis, but residual confounding and heterogeneity prevent it from being considered a stand-alone chemopreventive strategy (7, 25, 33). Proposed mechanisms include reduction of oxidative stress, cyclooxygenase modulation, and peroxisome proliferator-activated receptor gamma (PPAR-γ) signaling. 5-ASA may be continued when clinically indicated, but it does not replace inflammation control, surveillance, or dysplasia management.

Thiopurines can maintain IBD remission but are associated with established risks of lymphoma and non-melanoma skin cancer, and evidence for CAC chemoprevention is inconsistent (25, 33, 74, 85, 86, 110, 111). In patients with active or recent cancer, decisions to continue or restart azathioprine or 6-mercaptopurine should consider cancer type, recurrence risk, prior exposure, alternatives, and the consequences of uncontrolled IBD. A universal recommendation to prioritize another drug class is not supported for every clinical scenario.

### 6.2 TNF antagonists: therapeutic dichotomy and clinical considerations

Tumor necrosis factor (TNF) antagonists reduce inflammatory burden and, in mouse models, can alter the microbiota and attenuate inflammation-associated colorectal tumorigenesis (53). These experiments do not establish a direct antitumor effect in patients. Available clinical evidence has not demonstrated a uniform increase in new or recurrent cancer with TNF antagonists, but selection bias, limited follow-up, and underrepresentation of active cancer constrain inference (74, 85, 86, 110, 111). Treatment should therefore be individualized rather than based on a presumed class-wide pro- or antitumor effect.

Current guidance does not support a universal two-year prohibition on anti-TNF therapy after cancer diagnosis. Decisions should be individualized through multidisciplinary discussion according to cancer type and stage, time since treatment, recurrence risk, IBD activity, and available gut-selective alternatives (11, 16, 66, 74, 86, 110, 111). Active, uncontrolled inflammation also carries harm; therefore, the balance between disease control and oncological risk should be revisited over time.

### 6.3 Emerging therapies: IL-12/IL-23 inhibitors and JAK inhibitors

Newer biologics and small-molecule drugs expand treatment options, but comparative safety evidence in patients with active or recent cancer remains limited (74, 85, 86, 110, 111). Choice of agent should be based on IBD phenotype, prior response, cancer history, treatment interactions, and the strength of indication rather than an assumed oncological advantage.

Ustekinumab and IL-23 p19 inhibitors have not shown a strong malignancy signal in available IBD safety data, but patients with recent or active cancer are underrepresented in pivotal trials (29, 77, 86, 110). These agents are targeted immunomodulators rather than evidence-based CAC “memory” therapies. Treatment in patients with cancer should remain individualized, with acknowledgement of limited long-term, cancer-specific comparative data.

JAK inhibitors block cytokine signaling, including pathways converging on STAT3, and their short half-lives can facilitate perioperative management. However, direct reversal of epigenetic inflammatory memory in human CAC has not been demonstrated (57, 58, 111). Age, cardiovascular and thrombotic risk, infection risk, prior malignancy, and concomitant cancer therapy must be considered; pharmacokinetic convenience should not be interpreted as superior oncological safety.

### 6.4 Immune checkpoint inhibitors: the dilemma between efficacy and IBD exacerbation

Immune checkpoint inhibitors (ICIs) are most relevant to mismatch-repair-deficient/microsatellite-instability-high colorectal cancers. Patients with pre-existing IBD can benefit, but retrospective cohorts show an increased risk of gastrointestinal immune-related adverse events and IBD flare (112, 113). The decision requires oncological indication, baseline IBD activity, prior surgery, and a coordinated monitoring plan.

Vedolizumab and other selective immunosuppressive approaches are used to treat checkpoint-inhibitor colitis and may help manage IBD during cancer therapy, but evidence does not support blanket prophylactic use for every patient (110, 112, 113). Early stool testing, endoscopy when indicated, infection exclusion, and graded treatment according to severity are preferable to assuming that gut-selective therapy cannot influence antitumor efficacy.

Microbiome composition may correlate with ICI response, yet probiotic supplementation—including *A. muciniphila*—is not a validated strategy for patients with IBD and CRC (81, 109). Clinical decisions should therefore prioritize approved oncological and IBD therapies, multidisciplinary monitoring, and trial enrollment rather than unstandardized microbial products.

## 7 Conclusion and future perspectives

### 7.1 Pathogenesis of colitis-associated cancer

CAC arises through interactions among repeated injury, genetic selection, epigenetic persistence, immune remodeling, microbial metabolism, and tissue mechanics (88). No single component is sufficient in every patient. The inflammatory-memory framework is useful when it generates testable questions about persistence after signal withdrawal, the cell or compartment carrying the state, its functional consequence, and its reversibility.

AP-1-associated enhancer accessibility provides an important experimental example, but histone acetylation and methylation, DNA methylation, Polycomb regulation, enhancer transcription, and higher-order chromatin organization must be considered together (14, 59, 78, 97, 101, 103–105). Similarly, epithelial plasticity may enlarge the pool of tumor-initiating cells without establishing a single universal non-LGR5 origin (36, 99).

### 7.2 Conflicting data, model limitations, and unresolved questions

Persistent inflammatory programs can promote tissue repair as well as tumorigenesis. Their net effect depends on timing, dose, cell type, mutation status, microbial context, and whether resolution mechanisms remain intact. *A. muciniphila*, IL-22, macrophage polarization, Treg activity, neutrophils, and fibroblast remodeling all illustrate this stage dependence. A finding in active colitis cannot automatically be assigned the same function in remission, dysplasia, or invasive cancer.

AOM/DSS models combine chemical mutagenesis with acute cycles of injury and are valuable for intervention testing but do not reproduce decades of human IBD. Il10−/− and genetically engineered models capture different immune or epithelial mechanisms; organoids preserve epithelial cell-intrinsic features but usually lack vasculature, microbiota, immune cells, and mature stroma (37, 71). Human cross-sectional tissues demonstrate association and spatial coexistence, whereas persistence, reversibility, and causality require serial sampling or perturbation. Throughout this review, claims are therefore labeled as human, animal, organoid, or *in vitro* evidence where translation might otherwise be overstated.

### 7.3 From signal interception to selective memory modulation

Future chromatin-directed approaches include BET/BRD4 and histone deacetylase (HDAC) inhibitors and locus-specific CRISPR-based epigenetic editing (14, 59, 78, 97, 98, 106). At present, relevant CAC evidence is preclinical or proof-of-concept. Broad chromatin drugs may impair physiological repair, hematopoiesis, or immunity, whereas CRISPR epigenome editing faces delivery, specificity, reversibility, and germline-safety challenges. The goal should therefore be selective, time-limited modulation of pathological persistence rather than indiscriminate “memory erasure”.

### 7.4 Precision microenvironmental and microbiome editing

Tungstate, bacteriophage therapy, engineered probiotics, and defined microbial products offer complementary ways to alter the tumor-promoting niche (54, 107, 108). Their promise must be balanced against ecological instability, strain-specific effects, resistance, gene transfer, manufacturing, and host safety. The bidirectional evidence for *A. muciniphila* illustrates why microbial interventions require stage-specific biomarkers and randomized human trials rather than species-level assumptions (81, 100, 109).

Precision prevention will likely combine control of mucosal inflammation, high-quality surveillance, validated molecular risk markers, and carefully staged experimental interventions. Longitudinal human sampling linked to organoids, single-cell and spatial multi-omics, and functional perturbation will be needed to determine which persistent states are causal, reversible, and safe to target.

## Data Availability

The original contributions presented in the study are included in the article/supplementary material. Further inquiries can be directed to the corresponding author.
